# Effects of Low-Dose Diethylstilbestrol Exposure on DNA Methylation in Mouse Spermatocytes

**DOI:** 10.1371/journal.pone.0143143

**Published:** 2015-11-20

**Authors:** Li Yin, Li-juan Zheng, Xiao Jiang, Wen-bin Liu, Fei Han, Jia Cao, Jin-yi Liu

**Affiliations:** 1 Institute of Toxicology, College of Preventive Medicine, Third Military Medical University, Chongqing, China; 2 Gansu People’s Hospital, Lanzhou, China; Meharry Medical College, UNITED STATES

## Abstract

Evidence from previous studies suggests that the male reproductive system can be disrupted by fetal or neonatal exposure to diethylstilbestrol (DES). However, the molecular basis for this effect remains unclear. To evaluate the effects of DES on mouse spermatocytes and to explore its potential mechanism of action, the levels of DNA methyltransferases (DNMTs) and DNA methylation induced by DES were detected. The results showed that low doses of DES inhibited cell proliferation and cell cycle progression and induced apoptosis in GC-2 cells, an immortalized mouse pachytene spermatocyte-derived cell line, which reproduces primary cells responses to E2. Furthermore, global DNA methylation levels were increased and the expression levels of DNMTs were altered in DES-treated GC-2 cells. A total of 141 differentially methylated DNA sites were detected by microarray analysis. Rxra, an important component of the retinoic acid signaling pathway, and mybph, a RhoA pathway-related protein, were found to be hypermethylated, and Prkcd, an apoptosis-related protein, was hypomethylated. These results showed that low-dose DES was toxic to spermatocytes and that DNMT expression and DNA methylation were altered in DES-exposed cells. Taken together, these data demonstrate that DNA methylation likely plays an important role in mediating DES-induced spermatocyte toxicity *in vitro*.

## Introduction

Diethylstilbestrol (DES) is an active synthetic estrogen that was used to prevent miscarriage and premature deliveries between 1947–1971[1]. Almost immediately after uterine dysfunction and reproductive tissue cancers were discovered in young individuals exposed to DES *in utero*, the U.S. Food and Drug Administration (FDA) banned its use for pregnancy support[2]. Nevertheless, DES continues to be used to treat prostate and breast cancers[3]. It has also been used as a feed supplement or subcutaneous implantation for cattle, sheep, and poultry to improve weight gain and produce leaner meat. As a result, it was present as a contaminant in food sources for years after the FDA banned its use in humans[4]. In addition to exposure to DES through its usage as a drug and ingestion of residues present in food, individuals may potentially be exposed to this compound during its manufacture and during product formulation. The concentration of DES in ambient air samples obtained from plants involved in its manufacture has ranged from 0.02 to 24 μg/m^3^[5].

In recent years, the significant relationship between low-level DES exposure and toxic effects has attracted the attention of many researchers. Large numbers of studies have demonstrated that *in utero* and/or neonatal exposure to DES decreases the fertility of adult males and male rodents[6, 7] by causing morphological alterations of the genital tract, including cryptorchidism, hypospadias, seminal vesicle and testis alterations, and impaired spermatogenesis[7–10]. Cryptorchidism is the absence of one or both testes from the scrotum. Hypospadias refers to a birth defect of the urethra in the male where the urinary opening is not at the usual location on the head of the penis. These developmental abnormalities in the male reproductive tract induced by DES is a result of DES’ estrogen effect. Current epidemiological investigation and laboratory research indicated that DES exerts its estrogen effects mainly through classical ER(estrogen receptor) signaling[11]. Nevertheless, the level of DES exposure assessed in the majority of these studies was a high dose—-10^−5^ M[12, 13], and few reports have considered low-dose DES exposure and its effects on reproductive toxicity.

In molecular mechanistic studies, genetic and epigenetic pathways have been implicated in DES-induced reproductive developmental abnormalities[14, 15]. As a well-characterized epigenetic modification, DNA methylation is important for gene regulation, transcriptional silencing, development, and tumorigenesis[16]. The methylation of genomic DNA is catalyzed by DNA methyltransferases (DNMTs), including Dnmt1, Dnmt3a, and Dnmt3b. Dnmt1 is the primary enzyme responsible for maintenance of DNA methylation patterns during DNA synthesis, and Dnmt3a and Dnmt3b function as *de novo* enzymes during development[17]. Abnormal DNMT expression has been associated with DNA hypomethylation and hypermethylation, which could lead to aberrant genomic responses and ultimately, to altered cellular functioning[18]. Sato *et al*. have reported that perinatal DES exposure alters DNMT expression and DNA methylation in the mouse uterus, leading to the development of vaginal clear cell adenocarcinoma[19]. A study of another neonatal mouse model of DES exposure has indicated that the expression of Dnmt3a and methylation of some genes are altered in the mouse seminal vesicle[20]. Moreover, some researchers have revealed that gestational DES exposure affects cardiac structure/function in adult male mice and leads to increases in cardiac Dnmt3a expression and DNA methylation in the CpG island within the calsequestrin 2 promoter in the heart[21].

Given the evidence that DES alters the developmental programming of spermatogenesis and induces changes in epigenetic modification as a possible mechanism underlying DES-induced diseases, the aim of this study was to examine the effects and mechanism of low-dose DES exposure on DNA methylation in spermatocytes. To this end, mouse spermatocyte-derived GC-2 cells were exposed to 2×10^−7^~2×10^−5^ M DES, and changes in global DNA methylation and DNMT expression were assessed. Furthermore, differentially methylated genes were screened using microarray analyses and confirmed by methylation-specific PCR (MSP).

## Materials and Methods

### 1 Materials

DES was purchased from Santa Cruz Biotechnology, Inc. (CA, USA), diluted in DMSO(dimethylsulfoxide) to 500 M and stored at -20°C. The final concentrations used here were 2×10^−7^, 2×10^−6^, and 2×10^−5^ M, and they were freshly diluted with DMEM to their final concentrations. Controls were treated with the same amount of DMSO (0.04%) used in the corresponding experiment. A TRIzol Reagent Kit was obtained from Invitrogen (Carlsbad, CA, USA), and a PrimeScript RT Reagent Kit was purchased from Takara (Otsu, Japan). GoTaq Hot Start Green Master Mix was obtained from Promega (Wisconsin, USA). Dnmt1, Dnmt3a, and Dnmt3b antibodies were obtained from Santa Cruz Biotechnology, Inc. An HRP-conjugated(horseradish peroxidase-conjugated) secondary antibody, an enhanced chemiluminescence kit and an Annexin V–FITC(fluorescein isothiocyanate) Apoptosis Detection Kit were purchased from Beyotime (Shanghai, China). An EDU(5-Ethynyl -2’- deoxyuridine) Cell Proliferation Kit was obtained from Ribo (Guangzhou, China), and an EZ DNA Methylation-Gold Kit was purchased from Zymo Research (Orange, CA, USA).

### 2 Cell Culture

Mouse spermatocyte-derived GC-2 cells were purchased from the American Tissue Culture Collection (ATCC, Rockville, MD, USA). Cells were grown in DMEM (dulbecco's modified eagle medium) high-glucose medium (Hyclone, Logan, UT, USA) supplemented with 10% FBS (fetal bovine serum) (Sijiqing, Hangzhou, China), 100 units/ml penicillin and 100 μg/ml streptomycin. Cultures were maintained in a humidified atmosphere with 5% CO_2_ at 37°C.

### 3 Cytotoxicity

The viability of GC-2 cells after DES treatment was examined by CCK8 (cell counting kit-8) assay. GC-2 cells were seeded in 96-well plates and grown in DMEM with 10% FBS at a density of 4000 cells/well. After cells were synchronized by growth in DMEM without FBS for 18 h, they were treated with various concentrations of DES (0~10^-4^M) for 24, 48, or 72 h, and 100 μl CCK8 solution (diluted in DMEM) was added to each well. Cells treated with DMSO without sinulariolide were used as blank control cells. The plates were then incubated at 37°C for 1 h, and optical density (OD) was measured at 450 nm using a microtiter ELISA (enzyme-linked immuno sorbent assay) reader (Bio-Rad, Hercules, CA), with DMSO used as a blank. All experiments were repeated four times.

### 4 Cell Proliferation

GC-2 cells cultured in 96-well plates were treated with 2×10^−7^, 2×10^−6^, or 2×10^-5^M DES for 48 h, and DMSO was added to control cells. Cell proliferation was assessed according to the EDU Cell Proliferation Kit manual. Cells were photographed under a fluorescence microscope (OlympusCK40-32PA, Chinetek Scientific, Hong Kong, China).

### 5 Cell Cycle

GC-2 cells were seeded in 60 mm plates and grown in DMEM with 10% FBS at a density of 4.3 × 10^3^ cells/cm^2^. They were then treated with various concentrations of DES (0.2, 2, or 20 μM) or 0.04% DMSO for 48 h. All treated and control cells were fixed with ice-cold 75% ethanol for 24 h and were then stained, following the cell cycle kit protocol.

### 6 Apoptosis Evaluation by Flow Cytometry and Hoechst 33258 Staining

To examine DES-induced apoptosis in GC-2 cells, an Annexin V–FITC Apoptosis Detection Kit and Hoechst 33258 stain were used to assess the apoptosis rate and cell morphology. Flow cytometry was performed according to the Annexin V–FITC Kit protocol. Cells used in morphology analysis were fixed with 4% paraformaldehyde in PBS solution for 10 min, washed with PBS and then stained with Hoechst 33258 for 10 min at 37°C.

### 7 RNA Extraction and Real-Time PCR

Total RNA was extracted from GC-2 cells using a TRIzol Reagent Kit. Reverse transcription was performed using a PrimeScript RT Reagent Kit with gDNA Eraser (Promega, Madison, WI, USA). The mRNA expression levels of rxra, mybph, and prkcd were determined by real-time PCR, as in our previous study[22]. Primers were designed using the pubmed database. Real-time PCR was performed with a iQ™5 Real-Time PCR Detection System (Bio-Rad, Hercules, CA, USA), using the SYBR Green I detection method. Fold induction was normalized to GAPDH expression.

### 8 Western Blot Analysis

The treated and control samples (80 μg) were separated by 10% SDS-PAGE and then transferred onto a PVDF membrane for 1.5 h at 100 V. The membranes were incubated with 5% dehydrated skim milk to block nonspecific protein binding and then incubated with primary antibodies against Dnmt1, Dnmt3a, and Dnmt3b at 4°C overnight. An HRP-conjugated secondary antibody was added, and the membranes were incubated for 1 h at room temperature. Finally, they were visualized using an enhanced chemiluminescence kit.

### 9 Analysis of Global DNA Methylation

Total genomic DNA was extracted from GC-2 cells using a DNA Isolation Kit. The genome-wide methylation level was detected by 5-Methylcytosine DNA blot hybridization following the manufacturer’s protocol. The mouse Anti-5-Methylcytosine monoclonal antibody has been developed to facilitate differentiation between methylated and non-methylated cytosines in DNA.

### 10 Microarrays Analysis

Gene promoter methylation was analyzed using an Affymetrix Mouse Promoter 1.0R Array. Microarray experiments and data analyses were performed by the Gminix Company (Shanghai, China). The original files were mapped to the chromosome hg19, and then we got the corresponding original data. Then by Loess standard method the raw data was standardized. And based on the standard data of experiment group and control group, we can calculate the ratio between experiment group and control group, the absolute methylation level and relative methylation levels of each promoter in each sample. We used the CMARRT algorithm to calculate the enrichment region of the probe peak, the value p was calculated by the Gauss distribution. And then the promoter region of the gene was annotated, the annotation file was according to the hg19 database. Compare the differences in the methylation of excremental group and control group, and thus we obtained the difference between these groups[23]. The sites of differential DNA methylation with the highest fold changes were selected, and their respective results were confirmed by MSP (methylation-specific PCR) and real-time PCR.

### 11 Bisulfite Conversion and MSP

DNA was extracted from GC-2 cells using a DNA Isolation Kit and was chemically modified using an EZ DNA Methylation-Gold Kit (Zymo Research, Orange, CA, USA). Primer pairs that specifically amplified either methylated or unmethylated sequences spanning the CpG islands of selected genes were used for MSP, as detailed in S1 Table. MSP was carried out as in our previous studies[24].

### 12 Statistical Analysis

All results were expressed as the mean ± SE. Data were analyzed by one-way ANOVA (*P*<0.05). All univariate and regression analyses were performed using SPSS Software Package (Version 16.0, 2007).

## Results

### 1 Effects of DES on GC-2 Cell Viability and Proliferation

**Fig 1:**
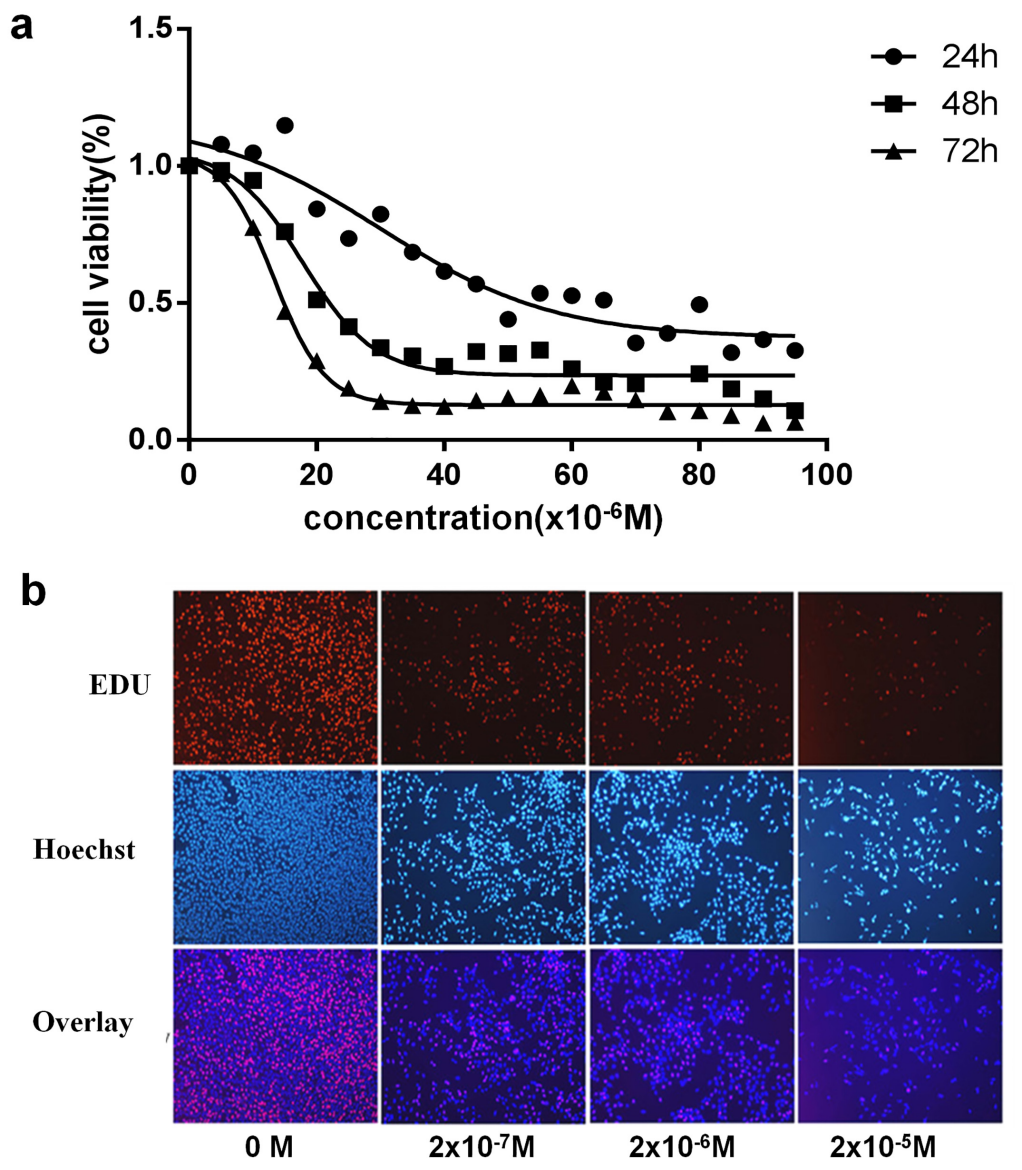
Effects of DES on GC-2 cell viability and proliferation. **a.** GC-2 cells were treated with 0~10^−4^ M DES for 24, 48 or 72 h. Cell viability was measured by CCK8 assay. **b.** GC-2 cells were treated with the indicated concentrations of DES for 48 h. The fluorescent thymidine analog EdU was used to identify GC-2 cells by the labeling of their DNA. Hoechst-labeled nuclei was shown in blue, and EdU-labeled newborn cells were shown in red.

To explore the potential cytotoxic effects of DES on GC-2 cells, cell proliferation was assessed. GC-2 cells were treated with various concentrations (0~10^−4^ M) of DES for 24 h, 48 h, or 72 h. As shown in Fig 1A, DES exposure clearly reduced the viability of GC-2 cells in a dose-dependent manner within a certain concentration and time range. In addition, cell proliferation was significantly decreased when cells were exposed to different DES concentrations. According to Fig 1B, the proportion of newborn (newly divided) cells decreased with increasing DES concentrations, indicating that the DNA replication capacity of GC-2 cells was decreased following DES exposure. Notably, even at a DES concentration of as low as 2×10^−7^ M, the proportion of newborn cells was lower than that in the DMSO group, suggesting that low doses of DES had adverse effects on mouse spermatocytes.

### 2 Effect of DES on GC-2 Cell Cycle Progression

**Fig 2:**
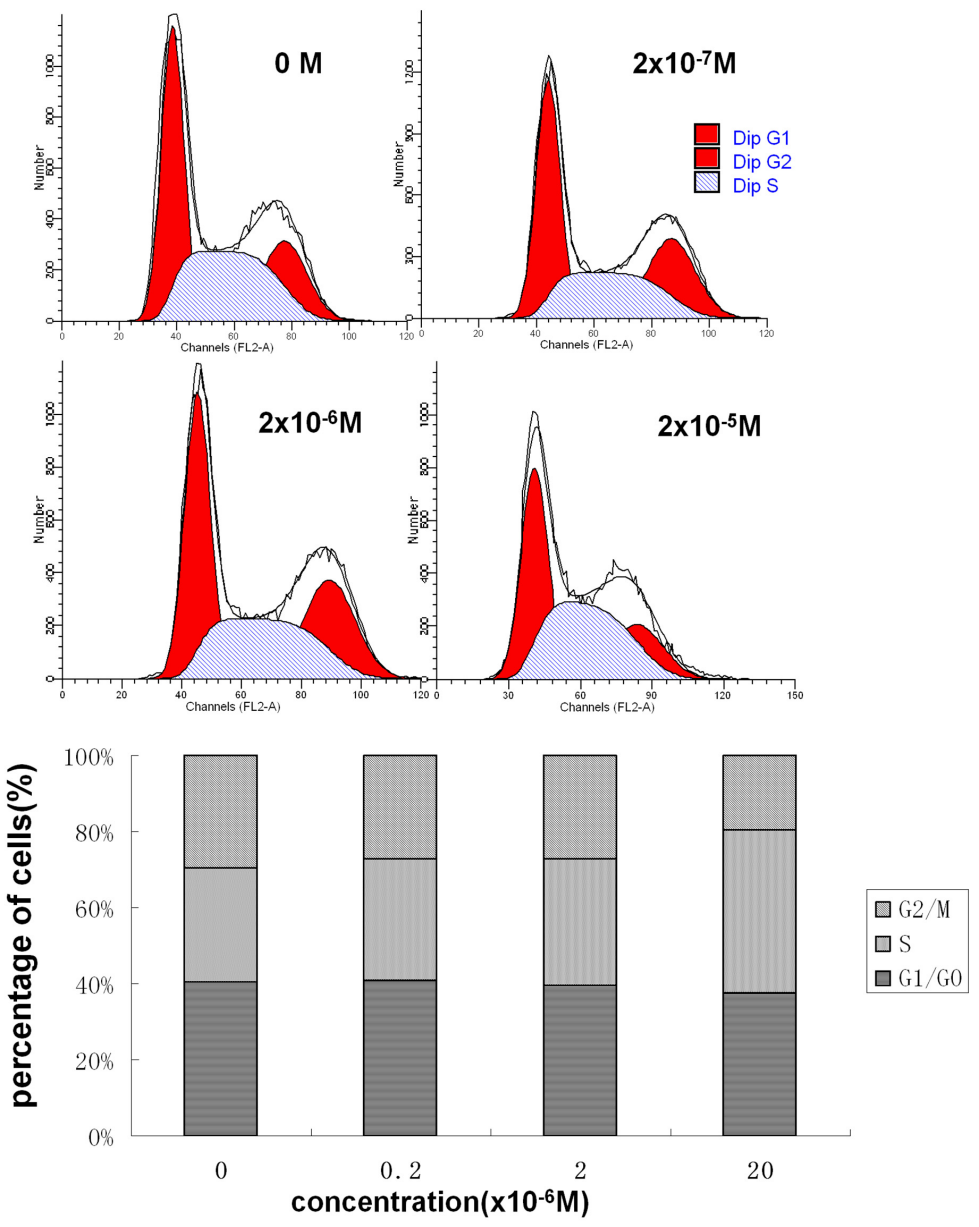
Effect of DES on GC-2 cell cycle progression. GC-2 cells were treated with the indicated concentrations of DES for 48 h. Cell cycle distribution was assessed by the propidium iodide method using flow cytometry.

Cell cycle progression is an important factor influencing cell proliferation. We analyzed the influence of DES on cell cycle progression to evaluate its antiproliferative activity. The proportion of cells in S phase increased for the DES-exposed cells compared with that for the DMSO-exposed cells (Fig 2). In particular, the proportion of S phase cells for the DMSO group was 28.97±1.21%, while those for the 2×10^−7^, 2×10^−6^, and 2×10^−5^ M DES-treated groups were 33.72±3.35%, 35.68±3.50%, and 43.44±3.65%, respectively. These results showed that DES changed the proportion of cells in the S cell phase and affected GC-2 cell cell cycle progression.

### 3 DES Induced Apoptosis in GC-2 Cells

**Fig 3:**
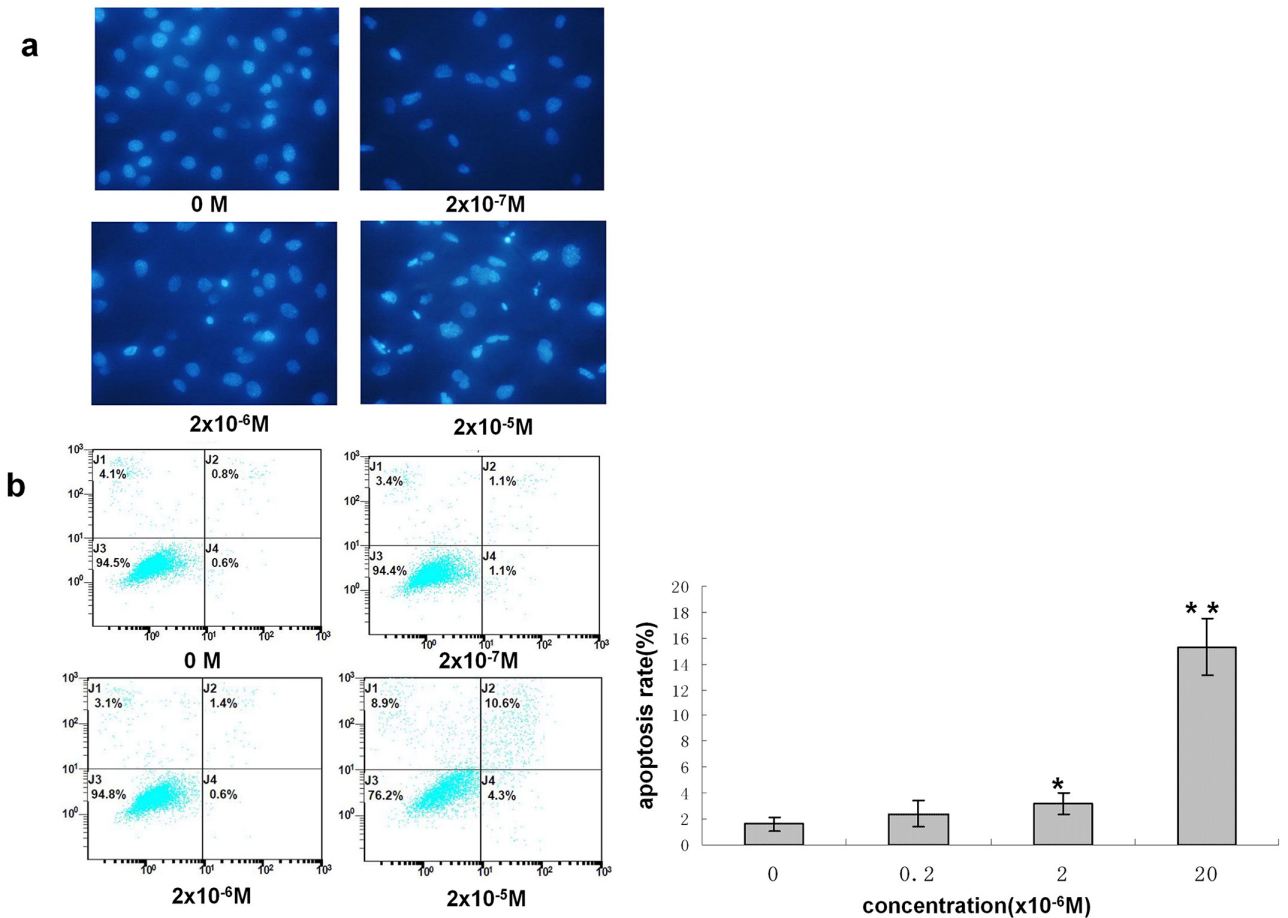
DES induces apoptosis in GC-2 cells. **a.** GC-2 cells were treated with the indicated concentrations of DES for 48 h. Apoptosis assay was also carried out using Hoechst 33258 staining. **b.** GC-2 cells were treated with the indicated DES concentrations for 48 h. Apoptosis assay was performed using flow cytometry after Annexin V-FITC/PI staining. Viable cells are shown in the lower left quadrant, early apoptotic cells are shown in the lower right quadrant, late apoptotic and necrotic cells are presented in the upper right quadrant, and nonviable necrotic cells are shown in the upper left quadrant. The data represent the mean ± SD; *P<0.05 and **P<0.01 compared with the DMSO-treated group.

Apoptosis is another important factor contributing to cell proliferation. Thus, we further examined apoptosis in GC-2 cells treated with or without DES. Apoptotic cells were recognized by their fragmented, degraded nuclei and apoptotic bodies. DES-treated GC-2 cells showed nucleolus pyknosis, and at increasing doses of DES, more nuclear fragmentation was observed (Fig 3A). Flow cytometric analysis also produced similar results (Fig 3B). After treatment with 0, 2×10^−7^, 2×10^−6^, and 2×10^−5^ M DES, the apoptosis rates were (1.3±0.52)%, (2.4±0.95)%, (2.7±0.68)%, and (16.8±1.34)%, respectively. Altogether, these results demonstrated that DES induced apoptosis in GC-2 cells.

### 4 Effects of DES on Global DNA Methylation in GC-2 Cells

**Fig 4:**
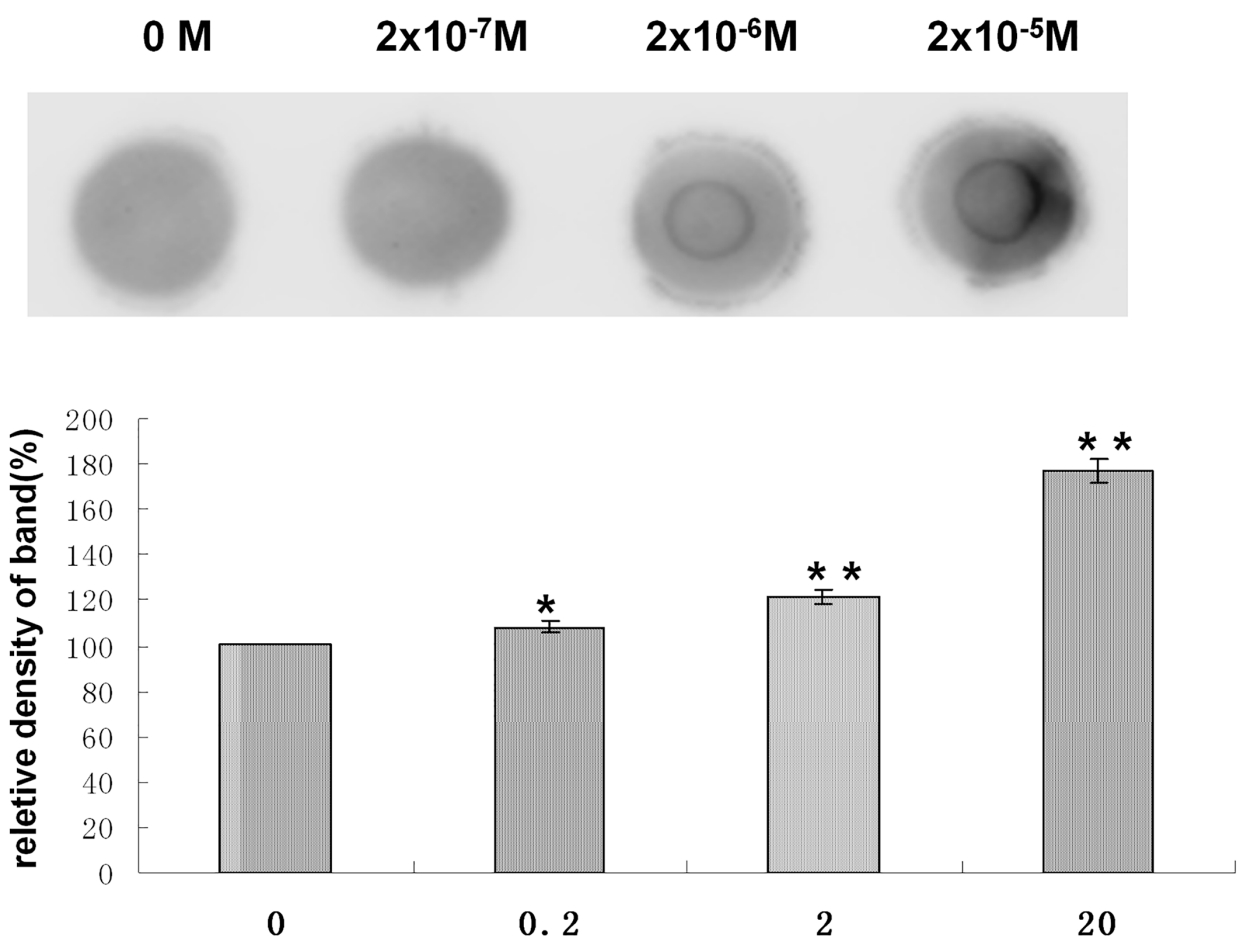
Effects of DES on global DNA methylation in GC-2 cells. The DNA 5-mC level was estimated by dot blot analysis. The gray values indicated DNA methylation levels.

Given the important effects of DNA methylation on gene regulation, transcriptional silencing and development, we sought to determine whether the DNA methylation patterns varied between GC-2 cells with and without DES exposure. We performed 5-mC dot blot DNA hybridizations to analyze the methylation statuses of GC-2 cells with and without exposure to this compound. Fig 4 showed the results of this analysis, with the density of band indicating relative DNA methylation levels, the global DNA methylation level in GC-2 cells was slightly increased following exposure to 2×10^−7^ and 2×10^−6^ M DES, and it was significantly increased in GC-2 cells exposed to 2×10^−5^ M DES. These data indicated that the global DNA methylation level increased with increasing concentrations of DES. They further suggested that DNA methylation might be crucial for the GC-2 cell toxicity observed following low-dose DES exposure.

### 5 Effects of DES on DNMT Expression

**Fig 5:**
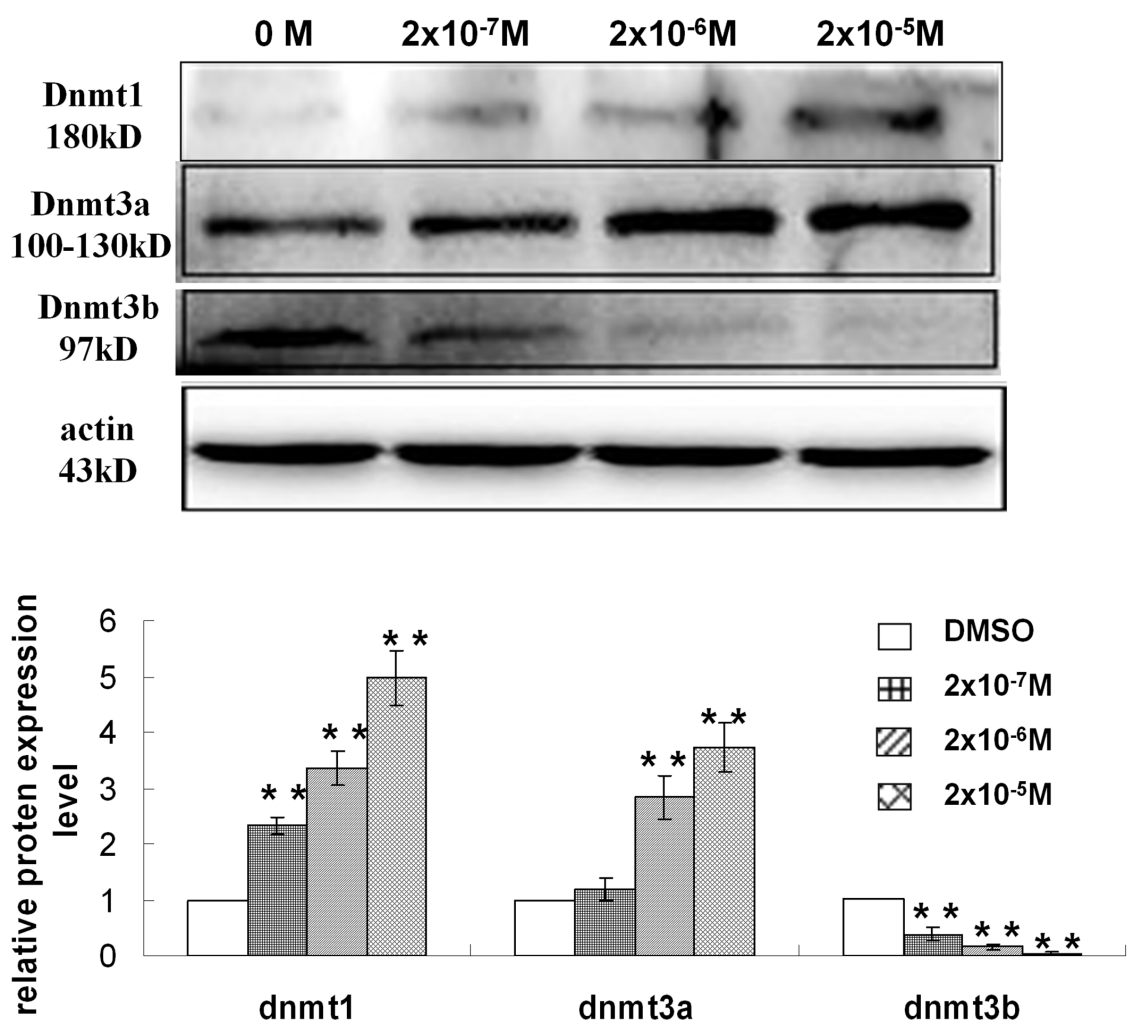
Effects of DES on the protein expression of DNMTs in GC-2 cells. *P<0.05 versus the DMSO-treated group, and **P<0.01 versus the DMSO-treated group.

Because DNMTs were found to play important roles in establishing and maintaining DNA methylation patterns, we determined the expression levels of Dnmt1, Dnmt3a and Dnmt3b. Compared with the control, Dnmt1 and Dnmt3a expression was slightly increased in GC-2 cells exposed to 2×10^−7^ M DES and significantly increased in cells exposed to 2×10^−6^ and 2×10^−5^ M DES. In contrast, Dnmt3b expression decreased sharply with increasing doses of DES (Fig 5).

### 6 Analysis of Differential DNA Methylation following DES Exposure

**Fig 6:**
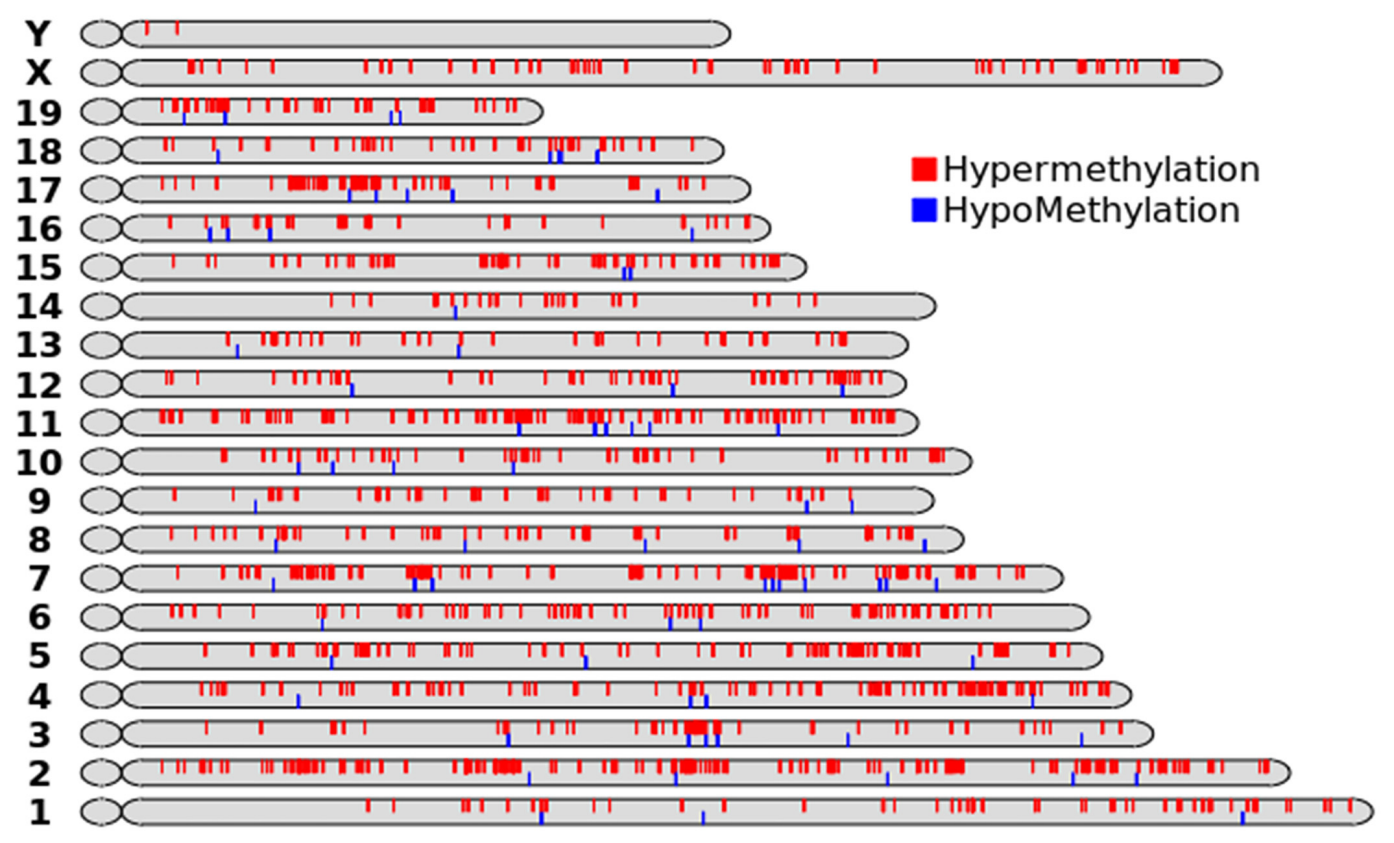
Chromosomal distributions of hypomethylated and hypermethylated genes in GC-2 cells exposed to 2×10^−5^ M DES. The red indicated the promoter of some genes was hypermethylation, and the blue showed that was hypomethylation in GC-2 cells were exposed to 2×10^−5^ M DES.

**Fig 7:**
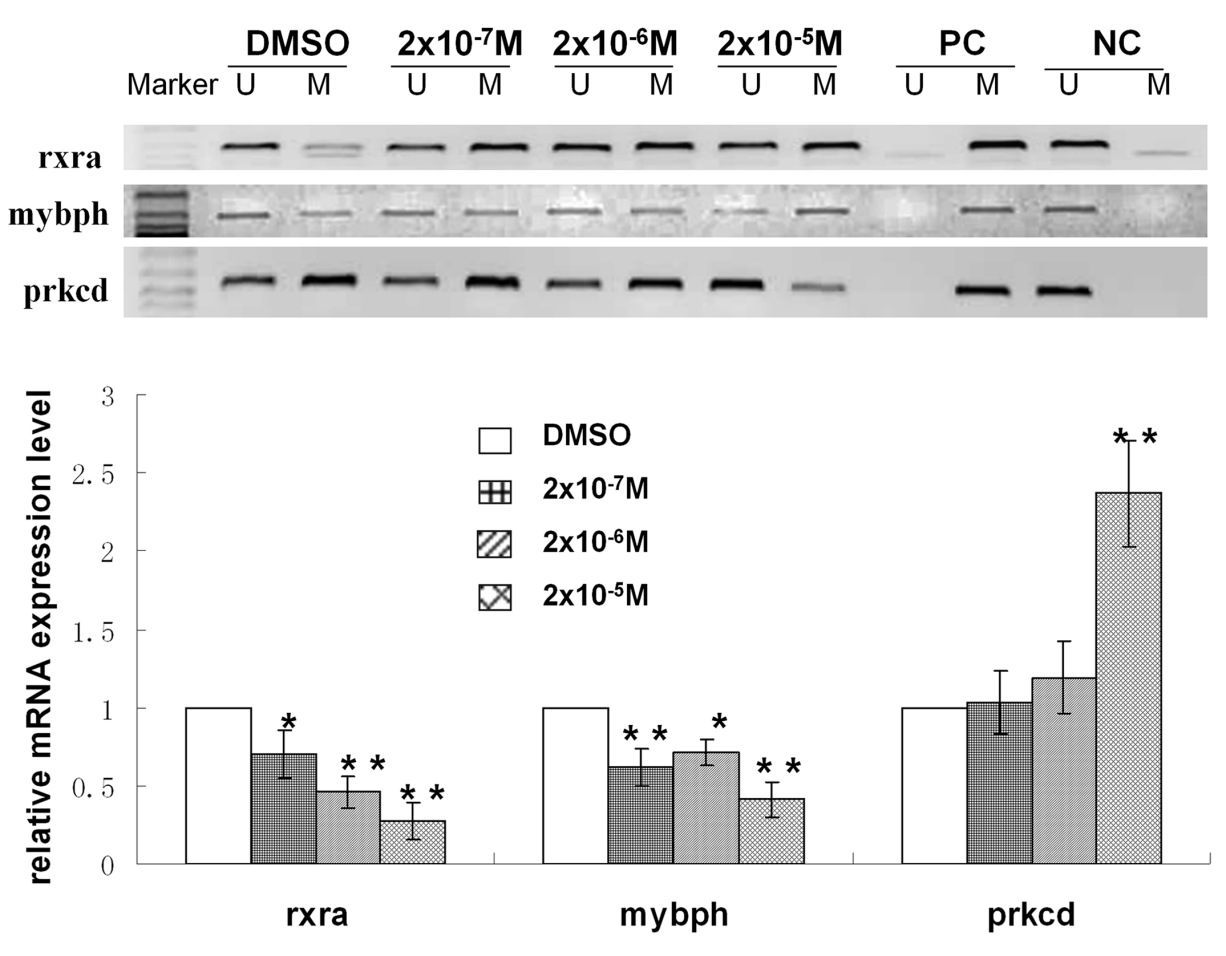
Effects of DES on the DNA methylation of rxra, mybph, prkcd. U, unmethylated-specific primers; M, methylation-specific primers; PC, positive control; NC, negative control. *P<0.05 versus the DMSO-treated group; and **P<0.01 versus the DMSO-treated group.

To further explore DES-induced alterations in methylation, we screened differentially methylated DNA sites using an Affymetrix Mouse Promoter 1.0R Array. The results of differential genome-wide methylation profiling of the DES-treated and control groups are shown in Fig 6. Data are available at GEO datasets (GEO number: GSE71311). A total of 141 differentially methylated sites (including 130 hypermethylated and 11 hypomethylated sites) were found in cells with and without 2×10^−5^ M DES exposure (fold change>3) by microarray analysis, some of which are listed in Table 1. As shown in Fig 7, the methylation statuses at some differential methylation sites were verified by MSP, and mRNA expression levels were detected by real-time PCR. In brief, compared with control cells, retinoid X receptor α (rxra) was hypermethylated in cells exposed to 2×10^−7^, 2×10^−6^, and 2×10^−5^ M DES and its mRNA expression was downregulated with increasing doses of DES. Myosin-binding protein H (mybph) was hypermethylated in cells exposed to 2×10^−5^ M DES, and its expression level was also reduced significantly. Protein kinase C *δ* (prkcd) was hypomethylated in cells exposed to 2×10^−5^ M DES, and its mRNA expression was increased. These results indicated that the methylation statuses of these genes were inversely correlated with their mRNA expression levels in DES-exposed GC-2 cells, suggesting that DNA methylation was involved in the regulation of mRNA expression in these cells.

**Table 1:** Microarray analysis of differentially methylated genes in GC-2 cells treated with 2×10^-5^M DES.

Position	Name	Description	Function	Fold change
Chr17	Rnf5	ring finger protein 5	cell survival[25] and autophagy[26]	4.0
Chr17	agpat1	1-acylglycerol-3-phosphate O-acyltransferase 1	myoblast differentiation[27]	4.0
Chr3	mnd1	meiotic nuclear division protein1	DNA repair in meiosis[28]	4.0
Chr5	otop1	otopetrin 1	regulation of cellular calcium[29]	3.7
Chr10	lrp1	low-density lipoprotein receptor-related protein 1	Cellular growth and cellular signaling[30]	3.5
Chr1	Mybph	myosin-binding protein H	Reduces cell motility, metastasis[31], and hypospadias development[32]	3.5
Chr12	hbp1	HMG box-containing protein 1	correlated with mitotic arrest in germ cells[33]	3.4
Chr14	Prkcd	protein kinase C, delta	Cell proliferation[34] and cell death[35]	3.4
ChrX	nono	Nono	transcription, RNA processing, and DNA double-strand break repair[36]	3.4
Chr9	dusp7	dual-specificity phosphatases 7	regulation of kinase (ERK) signaling[37]	3.4
Chr13	ccno	cyclin O	apoptosis, cell cycle progression, and DNA damage[38]	3.3
ChrX	cd99l2	CD99-related molecule CD99-like 2	inflammatory response[39]	3.2
Chr7	chp2	calcineurin B homologous protein isoform 2	cell growth and metastasis[40]	3.2
Chr3	cldn11	claudin 11	azoospermia[41]	3.3
Chr3	jtb	jumping translocation breakpoint	mitochondrial function, cell growth and cell death[42]	3.2
Chr16	mpv17l	mpv17 mitochondrial membrane protein-like	mitochondrial oxidative stress and apoptosis[43]	0.3
Chr9	nlrx1	nlr family member X1	prevents mitochondria-induced apoptosis[44]	3.2
Chr2	prex1	phosphatidylinositol-3,4,5-trisphosphate-dependent Rac exchange factor 1	cell motility[45]	3.3
Chr12	psen1	presenilin 1	mitochondrial structure[46]	3.1
Chr9	rhoa	ras homolog gene family, member A	proliferation[47]	3.0

## Discussion

The present study has provided several lines of evidence demonstrating that low doses of DES induce spermatocyte toxicity by triggering apoptosis, inhibiting proliferation, and affecting cell cycle progression. We have further found that DNA methylation might play an important role in DES-induced spermatocyte toxicity.

DES has long been known to affect the male reproductive system by causing alterations, such as reproductive organ dysplasia, and germ cell damage[3]. With regard to germ cells, abnormal spermatogenesis is the most common type of DES-induced effect. Some researchers have found that exposure of mice to 5 μg DES results in major morphological alterations to the testes, as reflected by the absence of germ cells in several tubules[7]. Another study has reported that this compound (1.0 mg/kg) induces spermatogenic apoptosis in adult male rats[48]. In our study, the apoptosis rate of GC-2 cells exposed to 2×10^−5^ M DES was significantly increased compared with that of DMSO-treated cells, and these results are in agreement with those of previous studies. GC-2 cell cycle progression was also altered following exposure to 2×10^−5^ M DES. Specifically, the percentage of DES-treated cells in the S phase of the cell cycle was greater than that of DMSO-treated cells, indicating that DES induced S phase arrest in spermatocytes. Further analysis using an EDU Cell Proliferation Kit indicated that the percentage of newborn cells was decreased following DES exposure, even at a DES concentration of as low as 2×10^−7^ M. EDU is readily incorporated into cellular DNA during DNA replication. Mammalian spermatogenesis is a unique process involving successive differentiation steps, including spermatogonial mitosis, spermatocyte meiosis and spermiogenesis. Each primary spermatocyte duplicates its DNA and subsequently undergoes meiosis I to produce two haploid secondary spermatocytes, which later divide once more into haploid spermatids[49]. Interestingly, EDU incorporated into DES-treated spermatocyte cells less frequently than untreated cells. Based on these data, we proposed that low doses of DES can cause spermatocyte toxicity.

DNA methylation has been implicated in the regulation of spermiogenesis[50]. DNA methylation at promoter regions plays a role in gene silencing, and during spermiogenesis, methylation occurs to silence retrotransposons and imprinted genes[51]. Therefore, we proposed that DNA methylation might be involved in DES-induced spermatocyte toxicity. First, we detected the genome-wide methylation statuses of GC-2 cells exposed to 2×10^−7^, 2×10^−6^, or 2×10^−5^ M DES and found a tendency of increased methylation in these cells, even following exposure to low doses of DES. DNMTs, including Dnmt1, Dnmt3a, and Dnmt3b, were found to be involved in DNA methylation. Dnmt1 is responsible for the maintenance of DNA methylation during DNA synthesis, and Dnmt1-deficient embryos have been shown to have 30% less genomic methylation than that found in embryos[52]. This phenomenon was also embodied in our experimental results. Dnmt1 protein expression was increased in GC-2 cells treated with 2×10^−7^, 2×10^−6^, or 2×10^−5^ M DES, consistent with the increase in the global DNA methylation level. Previous studies demonstrated that ERα could upregulate Dnmt1 expression by directly binding to the DNMT1 promoter region in ER-positive human breast cancer cells MFC-7 cells[53]. DES has strong estrogenic activity, can activate ERα, and increase the expression of Dnmt1, which is coincidence with our results. Dnmt3a and Dnmt3b are *de novo* enzymes that establish methylation patterns. Spermatogonia deficient in Dnmt3a and Dnmt3b display variations in methylation patterns at paternally imprinted regions, which may impair spermatogenesis to an extent[54, 55]. Our results showed that low doses of DES were toxic to spermatocytes *in vitro* and caused alterations in the Dnmt3a and Dnmt3b protein expression levels. Taken together, our results suggest that DNA methylation plays a role in low-dose DES-induced male reproductive toxicity[20].

To further explore the potential mechanism of action of DES, DNA microarray technology is a useful tool for mapping methylation changes at multiple CpG loci[56]. Microarray analysis performed in this study revealed the presence of thousands of variations in DNA methylation between GC-2 cells with and without DES exposure. The genes that were found to be differentially methylated are involved in the following processes: DNA repair, including mnd1 and nono; cell cycle progression, including hbp1 and ccno; apoptosis and proliferation, including rnf5, prkcd, jtb, nlrx1, mybph and rhoa; male reproductive development, including mybph, cldn11 and fkbp6; and other processes. The MSP assay results confirmed that the methylation statuses of some of the abovementioned genes were associated with low-dose DES-induced GC-2 cell toxicity. Rxra, an important component of the retinoic acid signaling pathway, is a key regulator of embryonic development and has been linked to several birth defects[57]. Rxra knockout animals showed an increase in apoptosis, resulting in abnormal morphogenesis during development, in addition to abnormal cell proliferation, cell differentiation, and cell death processes in adult differentiated tissues[58]. The two zinc fingers of the rxra DNA binding domain fold to form a single structural domain that consists of two perpendicularly oriented helices, which resembles the corresponding regions of ER[59]. What’s more, Angelika Rosenauer et al indicated that transient expression in ER-negative human breast cancer cells MDA-MB-231 of wild-type ER directly stimulated the transcriptional response to RA(retinoic acid). Importantly, this activation was greater than that obtained by transfection of RAR(RA receptor), RXR(retinoid X receptor), or RAR combined with RXR, and the DNA binding domain of ER plays a key role in the response to RA-induced transcription[60]. These researches suggested that ER had relation with rxra, and DES, as a strong ER agonist, had effect on the express of rxra. Mybph directly inhibits rock1 and plays important roles in cell motility and proliferation[31]. In our study, the rxra and mybph promoters were hypermethylated, and their mRNA levels were reduced in low-dose DES-treated GC-2 cells. Accordingly, the viability of DES-treated cells was decreased, suggesting that decreases in the mRNA levels of rxra and mybph due to hypermethylation played important roles in low-dose DES-induced GC-2 cell toxicity. Prkcd is involved in the regulation of a variety of cellular functions, including apoptosis and cell growth and differentiation. Its overexpression has been shown to induce phenotypic changes indicative of apoptosis in several cell types[61]. Our results indicated that prkcd was hypomethylated and that its high expression in DES-exposed cells was correlated with the increased apoptosis rate, similar to the previously reported theoretical results. These findings suggested that DNA methylation played an important role in low-dose DES-induced male reproductive toxicity.

As is known to all, DES has multigenerational effects. Some researches found that there is a high prevalence of hypospadias in the sons of women exposed to DES in utero[62]. A nationwide cohort study in collaboration with a French association of DES-exposed women showed that a significant proportion of boys born to DES daughters exhibited hypospadias with no other molecular defects identified. DES-induced changes in epigenetic background and alteration of DNA methylation could be significant factors in the susceptibility to disease development. Epigenetic changes in the some genes, transmitted through the DES daughter, could explain such a finding[10]. In our study, low dose of DES could change methylation status of many genes in GC-2 cells. Based on these, we deduced that low dose of DES could affect the methylation of germ cells in the same way, and many of the epigenetic changes would transmitted from father to grandson. Therefore, it is necessary to make further study related to low DES exposure and DNA methylation in germ cells.

In conclusion, our results showed that low doses of DES inhibited the proliferation of GC-2 cells, altered cell cycle progression, triggered cell apoptosis, and induced male reproductive toxicity. Through molecular studies, we have found that global DNA methylation and DNMT expression vary in DES–exposed GC-2 cells. Additionally, differentially methylated DNA sites were found in GC-2 cells treated with DES compared with those treated with DMSO. These results suggested that epigenetic modification might be a potential mechanism of low-dose DES-induced male reproductive toxicity.

## Supporting Information

S1 Table Primer sequences used for MSP and Real-Time PCR in this study. (DOC)
