# Supplementary material for: Effects of Low-Dose Diethylstilbestrol Exposure on DNA Methylation in Mouse Spermatocytes
Source: PLoS One. 2015 Nov 20;10(11):e0143143. doi: 10.1371/journal.pone.0143143 (PMC4654501; doi:10.1371/journal.pone.0143143)
Supplement: S1 Table — (DOC) [file pone.0143143.s001.doc]

**Table S1 Primer sequences used for MSP and Real-Time PCR** in this study.

| Gene |  | Primer sequence(5’-3’) | Length  (bp) |
| --- | --- | --- | --- |
| rxra | MSP(M) | Forward: GTATAAAAATAGTTTGGGGTTACGG | 120 |
|  |  | Reverse: AAATACTACTCGAAATACCCACGTT |  |
|  | MSP(U) | Forward: GTATAAAAATAGTTTGGGGTTATGG | 120 |
|  |  | Reverse: AAATACTACTCAAAATACCCACATT |  |
|  | Real-Time PCR | Forward: ACATTGTTGGGCGACTTTTGC | 184 |
|  |  | Reverse: TTTGGTGTCCATGTCTGCGA |  |
| mybph | MSP(M) | Forward: ATTGTGGGGATTAAGAGAGATAGC | 151 |
|  |  | Reverse: AAATCAAATCAAATCCATAAATCGA |  |
|  | MSP(U) | Forward: ATTGTGGGGATTAAGAGAGATAGTG | 150 |
|  |  | Reverse: AATCAAATCAAATCCATAAATCAAA |  |
|  | Real-Time PCR | Forward: GGACAAGGAGACAGCTTGGT | 119 |
|  |  | Reverse: GCCCCATTCGCTGTTCCTAT |  |
| prkcd | MSP(M) | Forward: GTTTTAGTTTGATTTTTTTGTTCGG | 223 |
|  |  | Reverse: TTACCGTTATTCTTCTTACAACGCT |  |
|  | MSP(U) | Forward: GTTTTAGTTTGATTTTTTTGTTTGG | 224 |
|  |  | Reverse: CTTACCATTATTCTTCTTACAACACT |  |
|  | Real-Time PCR | Forward: GAGCTCTTCGAGTCCATCCG | 221 |
|  |  | Reverse: GAAGGGGATTTCACTTTGGGC |  |
